# The Effect of Neurorehabilitation of the Cognitive Symptoms of Long COVID Evaluated with Neuropsi Atención y Memoria-III and BANFE-III

**DOI:** 10.3390/biomedicines13092267

**Published:** 2025-09-15

**Authors:** Ana Lilia Dotor-Llerena, Samuel Reyes-Long, Leilani Najera-García, Sandra Hernández-Corral, Elena Arechaga-Ocampo, Karina Avendaño-Ortiz, David Trejo-Martínez, Humberto Rosell-Becerril, Jose Luis Cortes-Altamirano, Alfonso Alfaro-Rodríguez

**Affiliations:** 1 Clinic Neuroscience, Instituto Nacional de Rehabilitación LGII, Mexico City 14389, Mexico; dotora_33@yahoo.com.mx (A.L.D.-L.); leilaninajera22@gmail.com (L.N.-G.); 2 Facultad de Estudios Superiores Zaragoza, Universidad Nacional Autónoma de México, Mexico City 09230, Mexico; hurbe00@gmail.com; 3 Basic Neurosciences, Instituto Nacional de Rehabilitación LGII, Mexico City 14389, Mexico; sam.long27@gmail.com (S.R.-L.); kariina.ortiiz06@gmail.com (K.A.-O.); drjlcortesaltamirano@gmail.com (J.L.C.-A.); 4 Departamento de Ciencias Naturales, Unidad Cuajimalpa, Universidad Autónoma Metropolitana, Mexico City 05348, Mexico; earechaga@cua.uam.mx; 5 Facultad de Enfermería y Obtetricia, Universidad Nacional Autónoma de México, Mexico City 14370, Mexico; scorral@inr.gob.mx; 6 Unidad de Investigación Multidisciplinaria en Salud, Instituto Nacional de Rehabilitación LGII, Mexico City 14389, Mexico; 7 Escuela Superior de Medicina, Instituto Politécnico Nacional, Mexico City 11340, Mexico; 8 Unidad de Neurocirugía Funcional, del Hospital General de México “Dr. Eduardo Liceaga” Secretaría de Salud, Mexico City 06720, Mexico; davidtrejomartinez@gmail.com; 9 Research Department, Universidad Estatal del Valle de Ecatepec, Ecatepec de Morelos 55210, Mexico

**Keywords:** long COVID, memory, attention, executive functioning, neuropsychology

## Abstract

**Background**: The long-haul symptoms of COVID-19 have not been properly attended, especially those of the central nervous system. Attention, memory and executive functioning are the three main cognitive symptoms reported for long COVID patients. To this day, neurorehabilitation therapy to alleviate these symptoms has not been proposed. **Objectives**: The current study aims to evaluate the effect of a neurorehabilitation intervention on the three most prevalent symptoms reported for long COVID in Mexican patients: memory, attention and executive functioning. **Methods**: Subjects were recruited at Instituto Nacional de Rehabilitación Luis Guillermo Ibarra Ibarra and underwent a novel neurorehabilitation intervention for 6 months. Baseline measurements were taken using validated instruments (Neuropsi, BANFE and CCQ) before the intervention and after it. **Results**: A significant decrease in the normalized score of the Memory component of the Neuropsi Atención y Memoria III test was found after the intervention, along with a decrease in two components of the BANFE-III test. **Conclusions**: In the current study, a successful neuropsychology intervention for the main cognitive symptoms of long COVID in a Mexican population reduced subjective self-perceived complaints and objectively measured cognitive symptoms.

## 1. Introduction

Amidst all of the consequences of the COVID-19 pandemic in several sectors of the population, the long-haul symptoms of this disease are some of the most relevant topics of research [1,2,3]. The hypotheses behind these symptoms are diverse; in particular, it has been proposed that cognitive or neuropsychological symptoms are caused by chronic neuroinflammation [4] or disruption of the blood–brain barrier [5]. Equally, in a recent study, it was found that the spike S protein of the virus was present across many systems, even after months of the infection, in an animal model of the disease [6], which could be a direct cause of its cognitive symptoms.

According to several systematic reviews [7,8], cognitive symptoms are amongst the most prevalent ones; these have been classified as memory impairments, attention problems and executive functioning disorders [9], commonly known as brain fog.

It is worth noticing that in spite of the extensive research on long COVID and its heavy burden on quality of life, the cognitive component of the disease has been neglected in comparison to anosmia, fatigue or pulmonary and cardiac symptoms. This can partially be explained by the lack of studies analyzing them from a neuropsychological standpoint using validated instruments and novel interventions.

The current study aims to evaluate the effect of a neurorehabilitation intervention on the three most prevalent symptoms reported for long COVID Mexican patients: memory, attention and executive functioning.

## 2. Materials and Methods

### 2.1. The Study Design and Participants

For this study, two groups were formed: (a) a control group of subjects that were diagnosed positive for COVID-19 by means of real-time reverse transcriptase polymerase chain reaction (RT-PCR) who were asymptomatic or whose symptoms did not continue after 3 months of the acute infection and (b) an experimental group of subjects that were diagnosed positive for COVID-19 using RT-PCR with self-reported memory or attention impairments that appeared after 3 months of the acute phase of SARS-CoV-2 infection.

Subjects aged 18 to 64 years old were recruited at the National Institute of Rehabilitation Luis Guillermo Ibarra Ibarra (INR-LGII), and the inclusion criteria were as follows: (1) at least one negative COVID-19 test from more than 3 months ago; (2) an education level > 9 years; and (3) at least 3 doses of vaccines against SARS-CoV-2. The exclusion criteria included (1) a family history of mild or severe neurocognitive impairments; (2) diagnosed psychiatric disorders previous to the SARS-CoV-2 infection; and (3) a diagnosis of autoimmune diseases or cancer. Elimination criteria included (1) attendance to the neurorehabilitation lower than 90% or (2) SARS-CoV-2 reinfection in the follow-up period.

This study was approved by the Ethics and Research Committee of INR-LGII with protocol number registration 03/23 and followed the guidelines outlined in the Declaration of Helsinki. All subjects voluntarily participated, signed their informed consent and were briefed on the aims and the follow-up period of the intervention. Moreover, the subjects were at liberty to quit the study at any time.

### 2.2. The Demographic Data and Neuropsychological Tests

Demographic and clinical data were collected by means of clinical history. All subjects underwent a thorough neuropsychological assessment that was performed by an expert in neuropsychology; the assessment comprised three validated instruments: the Cognitive Complaints Questionnaire (CCQ) [10]; a neuropsychological evaluation using Neuropsi Atención y Memoria III (NeuroPsi), an instrument designed to evaluate, in detail, control, selective and maintained attention, plus the types and stages of memory, including working memory and short-term and long-term memory [11]; and BANFE III (BANFE), a test designed to evaluate, in detail, executive functioning and the functionality of the frontal lobes [12]. Both tests possess normalized values for years of education and age. The assessment was performed at the baseline and 6 months later, when the neurorehabilitation had ended.

### 2.3. Intervention

For a period of 6 months, the subjects in the experimental group received an intervention through a program of neurorehabilitation that centered on attention to the cognitive symptoms reported for long COVID; the subjects had one in-person session a week of 1 h, and the attending neuropsychologist was the same one for every session. Additionally, in-house assignments were given to each subject; these were two sessions a week of 1 h each on a web platform named Neuron-Up. Memory and attention functions were intervened with in a consecutive manner, with a progressive increase in task difficulty and strategy complexity; meanwhile, executive functioning was considered the first transversal axis for promoting memory and attentional development, thus facilitating the identification and employment of compensatory strategies and ultimately fomenting the handling of external aids. In the same way, a second transversal axis was implemented: information processing speed. This was considered an epiphenomenon and a complement to all elements of the cognitive sphere that allowed us to identify the subject’s capacity to capture, comprehend and respond to the cognitive requirements.

**Figure 1:**
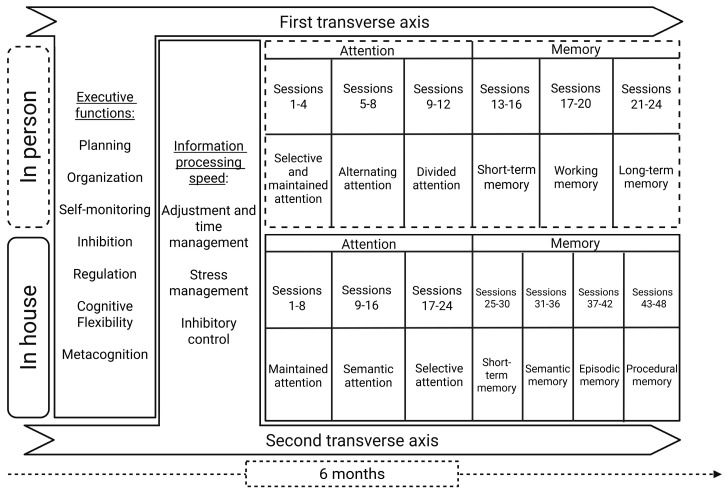
The organizational structure of the intervention model distributed in sessions.

The detailed structure of the neurorehabilitation intervention was as follows: attention was the first symptom that was intervened with, in its different modalities as sustained, selective, alternating and divided. Next, short-term, long-term, working, semantic, episodic and procedural memory at the codification, storage and evocation stages was intervened with. The two transversal axis were implemented in order to increase the attentional devices and the relationship between memory and executive functional processes, strengthening the employment and maintenance of cognitive strategies, as well as regulation and response time management, monitoring inhibitory control and stress (Figure 1).

### 2.4. Statistical Analysis

Descriptive statistics were employed for clinical and demographic data; its normal distribution was assessed using the Shapiro–Wilk test, and parametric statistics were employed. Qualitative data was expressed as proportions and quantitative data as the mean ± SD. The subjects’ scores from the two neuropsychological assessments were normalized with respect to their age and years of education. The normalized data was employed for a comparison between the controls and pre- and post-rehabilitation by means of a paired *t*-test, considering a significance level set at *p* < 0.05. GraphPad Prism 8.0.1 was employed for the data analysis.

## 3. Results

### 3.1. Clinical and Demographic Data

A total of 23 subjects were recruited for the experimental group; 2 subjects were excluded because they did not show 90% adherence to the intervention, 2 subjects contracted a respiratory infection throughout the course of the intervention and 1 was a patient with undetectable HIV, and thus, 18 subjects completed the neuropsychological rehabilitation overall. For the control group, a total of 15 subjects were recruited. The clinical and demographic data of both groups is displayed in Table 1. All subjects that were recruited went through the acute phase of SARS-CoV-2 infection with a mild condition, according to the WHO guidelines; no subjects required hospitalization, oxygen therapy or intubation.

The self-reported long COVID symptomatology of the experimental group subjects was as follows: fatigue: 14 (77.78%); headache: 7 (38.89%); attention problems: 18 (100.00%); hair loss: 10 (55.56%); shortness of breath: 2 (11.11%); sleeping problems: 11 (61.11%); articular pain: 6 (33.33%); generalized pain: 2 (11.11%); memory impairments: 14 (77.78%).

### 3.2. Neuropsychological Assessment Results

The general adherence of the experimental subjects to the intervention involved a total of 72 sessions, 24 distributed in person and 48 in-house sessions.

The results from the CCQ are presented in Table 2. The total score for each component of the questionnaire was averaged, and the SD was obtained; a statistically significant decrease was observed across all components: attention (*p* < 0.001), orientation (*p* = 0.04), executive function (*p* < 0.001), memory (*p* < 0.01), praxis and gnosis (*p* = 0.005), language (*p* = 0.001) and the total score in the questionnaire (*p* < 0.001). Also, in Table 2, the normalized values are presented; the statistical analysis was not performed with these values.

An objective neuropsychological diagnosis consisting of two tests was made before the neurorehabilitation intervention in the experimental group and only at recruitment for the control group. When evaluating the experimental group at the baseline using the neuropsychological battery Neuropsi Atención y Memoria III, the diagnosis of the population was as follows: Executive Functions: Normal: 11 (61.11%); Mild to Moderate Alteration: 3 (16.67%); Severe Alteration: 4 (22.22%). Memory: Normal: 12 (66.67%); Mild to Moderate Alteration: 2 (11.11%); Severe Alteration: 4 (22.22%). Total: Normal: 11 (61.11%); Mild to Moderate Alteration: 4 (22.22%); Severe Alteration: 3 (16.67%).

**Figure 2:**
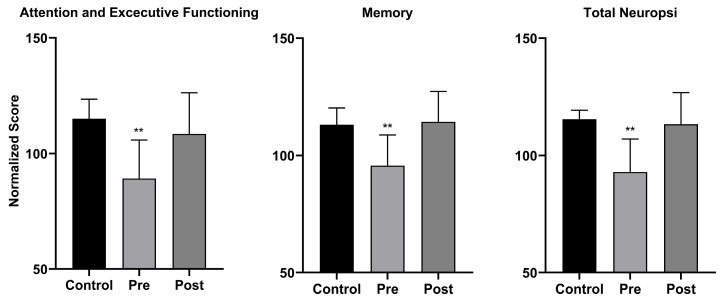
Normalized scores obtained in the Neuropsi Atención y Memoria III test comparing the control group and the experimental group pre- and post-neurorehabilitation (** *p* < 0.01).

In turn, to evaluate the effect of the neurorehabilitation intervention, after it ended, the Neuropsi Atención y Memoria III test was reapplied, and the normalized scores were compared to those obtained at the baseline. In this comparison, significant increases were found in two components of the test, Attention and Executive Functioning (*p* < 0.01) and Memory (*p* < 0.01), plus in the total normalized score (*p* < 0.01) (Figure 2). Significant differences were not found between the post-intervention scores of the experimental group and the control group.

Baseline diagnoses for the experimental group were obtained using the BANFE-III test. The results for the Orbitomedial component were as follows: Normal: 8 (44.44%); Mild to Moderate Alteration: 8 (44.44%); Severe Alteration: 2 (11.11%). In the Anterior Prefrontal component, they were as follows: Normal High: 2 (11.11%); Normal: 9 (50.00%); Mild to Moderate Alteration: 4 (22.22%); Severe Alteration: 3 (16.67%). In the Dorsolateral component, they were as follows: Normal: 8 (44.44%); Mild to Moderate Alteration: 2 (11.11%); Severe Alteration: 8 (44.44%). For the totals, they were as follows: Normal: 7 (38.89%); Mild to Moderate Alteration: 6 (33.33%); Severe Alteration: 5 (27.78%).

**Figure 3:**
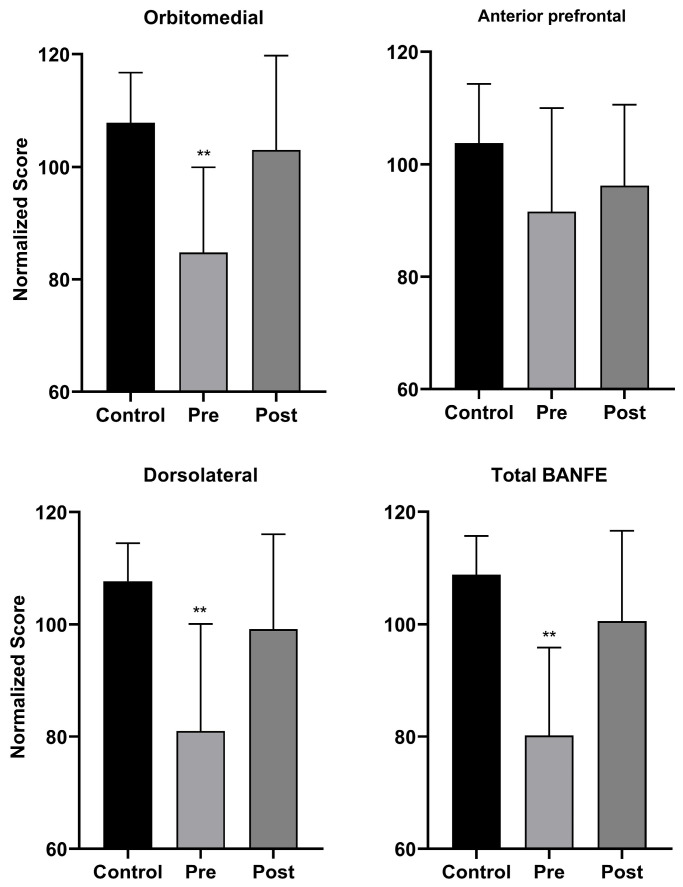
Normalized scores obtained from the BANFE-III test comparing the control group and the experimental group pre- and post-neurorehabilitation (** *p* < 0.01).

As above, at the end of the neurorehabilitation, BANFE-III was reapplied in the experimental group in order to evaluate the effect of the intervention. A significant increase was found in the normalized scores of both the Orbitomedial (*p* < 0.01) and Dorsolateral (*p* < 0.01) components and in the total normalized scores of the BANFE-III test (*p* < 0.01) (Figure 3). When comparing the post-neurorehabilitation scores for the anterior prefrontal cortex in the experimental group and the scores in the control group, no significant differences were found.

## 4. Discussion

In contrast with studies analyzing the etiology of cognitive symptoms in long COVID, the number studying interventions that could improve the people experiencing them have been few or even absent [13]. The present research was based on reports that the main neuropsychological alterations identified in people suffering from long COVID are memory impairments, attention deficits and problems with executive function [14]. This is in line with the results of the baseline neuropsychological evaluation in the present research, which allowed us to structure an intervention strategy focused on the rehabilitation of these cognitive functions.

Several studies have shown that long-term symptoms characteristic of long COVID are not correlated with the severity of the acute phase of the infection [15,16]; this was observed in the present study, as cognitive symptoms of long COVID in all subjects appeared after a mild course of COVID-19 according to the WHO classification.

In the present study, the Cognitive Complaints Questionnaire was applied to the participants in order to explore the perceptions they had about their own cognitive difficulties. Initially, mostly memory problems were reported; however, when contrasting this with the objective neuropsychological evaluations made by means of Neuropsi Atención y Memoria-III and BANFE-III, it was evident that their difficulties were focused on maintained and selective attention and executive function. This finding points to an expected discrepancy between the initial subjective perception of the participants and the deficits objectively measured using standardized tests.

Aware of this discrepancy, during the intervention, an integral approach was adopted which included neuropsychic education [17], allowing the subjects to identify and comprehend their own characteristics and functioning patterns. Through this strategy, self-knowledge and the subsequent implementation of personalized approaches that could positively impact cognitive functions were promoted. Not only was this process oriented towards an improvement in their quantitative evaluated capabilities but also metacognition was pursued, namely the capability to think about one’s own thinking processes [18], making it easier for the subjects to distinguish in a specific manner what their difficulties were and which strategies were more effective to compensate or substitute for them.

The results of the CCQ after the neurorehabilitation were highly revealing: a significant improvement in the self-perception of the subjects was observed, as they reached better levels than the normative data for healthy subjects [19]. This finding enables the hypothesis that neurorehabilitation not only improves quantitative cognitive measurements but also encourages an important qualitative development; the subjects became more self-aware of their cognitive difficulties and acquired the capacity to discern in a more specific manner which areas were affected and what compensatory strategies were more useful for them.

These findings highlight the impact of an integral neuropsychological approach in which the promotion of metacognition plays a crucial role; plus, the data from this study suggests that the neurorehabilitation interventions could benefit from the incorporation of specific modules focused on metacognitive development and this this could facilitate more effective and personalized adaptation to neuropsychological rehabilitation strategies [18,20,21].

The results of the present study indicate that their cognitive function in orientation was maintained between the normative ranges [10,22] in the neuropsychological evaluations, as well as in the CCQ. This contrasts with the other cognitive domains evaluated, where deficits or alterations were observed, thus allowing us to hypothesize that in the cognitive profile of long COVID patients, orientation can be maintained intact. This result is particularly relevant when comparing this pathology with other major neurocognitive disorders, in which it is common to observe significant alterations in the orientation component. The clinical implications of the above could be of great importance; the preservation of orientation in long COVID patients suggests that this particular function could be proposed as an early cognitive marker in screening protocols [23]. The incorporation of orientation-focused tests in the process of evaluation could allow for timely derivation of specialists, expediting the implementation of more adjusted rehabilitation protocols, which in turn could contribute to the optimization of resources and ultimately reduce the economic load on the health sector.

The results of the present study showed significant improvements in memory, attention and executive function, and a reduction in subjective cognitive complaints was also observed; clinical observation and interpretation allowed us to propose that memory is impacted by other cognitive domains like attention and concentration. In addition, these improvements did not directly affect visuospatial processing given that in the majority of cases, the qualitative performance in the complex figure test of Neuropsi Memoria y Atención III was the same pre- and post-neurorehabilitation. Paz-Rodríguez et al. [19] emphasize the importance of qualitative data because it allows for a multifactorial analysis that provides more fruitful data, opening the possibility of employing an integral evaluation of the visuospatial processing component as a putative prognostic marker of cognitive impairment, just as it has been employed in other pathologies, like Alzheimer’s, where reports show that these alterations appear up to 5 years earlier than other diagnostic elements of the disease [24].

The orbitofrontal regions, which are represented in the BANFE-III test, are implicated in emotional regulation and affective states [12,25]; plus, they also play a significant role in risk–benefit decision-making [26]. In the present study, a significant increase in the normalized scores for this component was found. This could be related to the implementation of strategies focused on executive functions as a transverse axis in the neurorehabilitation; in tasks aiming to strengthen attention and memory, strategies of memory flexibility, self-monitoring, regulation and verification were implemented in order to achieve a successful result. The absence of a statistically significance difference in the anterior prefrontal cortex assessed using the BANFE test could partially be explained by the fact that it considers subtests focused on abstract thinking skills, a cognitive process that is significantly influenced by level of education, taking into account crystallized knowledge, and as the number of years of education was an inclusion criterion, all subjects were at a similar academic level.

During the initial phase of the evaluation, some visuospatial difficulties were qualitatively observed in some of the subjects; while these are not particularly evident and are not considered fundamental in the literature, they were relevant in the day-to-day performance of the subjects of the present study. Paz-Rodríguez et al. [19] highlight the fact that recent research has focused only on three of the most reported symptoms, attention, memory and executive function, and has put aside visuospatial functions. Although they present with low consistency [27,28], visuospatial functions should be considered, as they play a significant role in day-to-day activities [29,30]. Given that visuospatial difficulties were not prioritized in the intervention, specific neurorehabilitation strategies were not implemented. The permanence of these visuospatial difficulties suggests that the functional impact on the subjects could not have been completely optimized, and this underlines the importance of continuing the design of interventions that consider a wider spectrum of cognitive functions.

The present study faced some limitations, like all studies with human subjects, with the time of the intervention being the most important; two of the subjects recruited did not fulfil the adherence criteria and had to be excluded, thus reducing the sample size.

## 5. Conclusions

In the present study, a novel and successful neurorehabilitation intervention is presented, as shown in the significant improvement in the cognitive symptoms of long COVID in a Mexican population, which is reflected as an increase in the objective values of neuropsychological batteries as well as a reduction in subjective self-perceived complaints. The neurorehabilitation intervention consisted of in-person and in-house sessions, which could be seen as both a limitation and as an opportunity; it reduced the costs and time invested in transport to the center of attention. However, the learning curve of the web-based platform had to be considered beforehand. Thus, the intervention proposed in the present study could be viable treatment option in the near future for patients that are still affected by the cognitive sequalae of COVID-19.

## Figures and Tables

**Table 1:** Clinical and demographic data of long COVID subjects.

Variable	Subjects (n = 18)	Controls (n = 15)
Sex		
Female	13 (72.22)	10 (66.67)
Male	5 (27.78)	5 (33.33)
Age (Mean ± SD)	47.94 ± 11.12	47.80 ± 10.09
Manual Dominance (Right-Handed)	17	15
Comorbidities		
Overweight	7 (38.89)	2 (13.33)
Diabetes Type 1	1 (5.55)	1 (6.67)
Hypertension	1 (5.55)	0
COVID-19 Symptoms		
Cough	9 (50.00)	5 (33.33)
Headache	13 (72.22)	9 (60.00)
Joint Pain	12 (66.67)	5 (33.33)
Anosmia	10 (55.56)	4 (26.67)
Ageusia	9 (50.00)	4 (29.67)
Diarrhea	5 (27.78)	2 (13.33)
Shortness of Breath	3 (16.67)	2 (13.33)
Generalized Pain	13 (72.22)	4 (26.67)
Chest Pain	5 (27.78)	7 (46.67)
Throat Pain	9 (50.00)	8 (53.33)

**Table 2:** The scores obtained in the Cognitive Complaints Questionnaire (CCQ).

Domain	Control	Pre	Post	*p*	Normative Values
Attention	2.04 ± 0.58	3.73 ± 0.53	2.79 ± 0.53	<0.001	4.73 ± 3.286
Orientation	1.33 ± 0.44	1.89 ± 0.49	1.46 ± 0.38	0.04	0.76 ± 1.048
Executive function	1.92 ± 0.82	2.85 ± 0.64	1.88 ± 0.58	<0.001	2.44 ± 2.892
Memory	1.83 ± 0.75	3.35 ± 0.63	2.21 ± 0.64	<0.001	4.13 ± 3.892
Praxis and gnosis	1.17 ± 0.20	2.42 ± 0.62	1.69 ± 0.67	0.005	1.66 ± 1.919
Language	1.42 ± 0.38	2.77 ± 0.91	1.88 ± 0.42	0.001	3.30 ± 2.856
Total	9.71 ± 2.75	17.00 ± 2.66	11.88 ± 2.024	<0.001	16.90 ± 11.948

Data is presented as mean ± SD.

## Data Availability

The original contributions presented in this study are included in the article. Further inquiries can be directed to the corresponding author.
